# Metabolomics and Lipidomics Reveal the Effect of Hepatic *Vps33b* Deficiency on Bile Acids and Lipids Metabolism

**DOI:** 10.3389/fphar.2019.00276

**Published:** 2019-03-22

**Authors:** Kaili Fu, Conghui Wang, Yue Gao, Shicheng Fan, Huizhen Zhang, Jiahong Sun, Yiming Jiang, Conghui Liu, Lihuan Guan, Junling Liu, Min Huang, Huichang Bi

**Affiliations:** ^1^School of Pharmaceutical Sciences, Sun Yat-sen University, Guangzhou, China; ^2^Department of Pathophysiology, Shanghai Jiao Tong University School of Medicine, Shanghai, China

**Keywords:** VPS33B, cholestasis, bile acids, metabolomics, lipidomics

## Abstract

Vascular protein sorting-associated protein 33B (VPS33B) plays important roles in hepatic polarity, which directly maintains the functional structure of the liver. It has reported that VPS33B has close association with arthrogryposis, renal dysfunction and cholestasis (ARC) syndrome. Unfortunately, no further studies were conducted to reveal the role of *Vps33b* in the homeostasis of bile acids. In the current study, hepatic *Vps33b*-depleted male mice were used to investigate the metabolomics and lipidomics profiles of hepatic *Vps33b* deficiency based on ultrahigh-performance liquid chromatography coupled with an electrospray ionization high-resolution mass spectrometry (UHPLC-ESI-HRMS) system. Hepatic *Vps33b*-depleted male mice displayed cholestasis and slight liver damage with increased serum levels of ALT, AST, ALP and T-Bili compared to wild-type mice. Targeted metabolomics analysis of bile acids revealed that increased taurine-conjugated bile acids accumulated in the serum of hepatic *Vps33b*-depleted mice, while unconjugated bile acids were prone to decrease, accompanied by the regulation of bile acid homeostasis-related genes. In addition, lipid profiles were significantly altered with the lack of *Vps33b* in the liver. A variety of lipids, such as triglycerides and sphingomyelins, were significantly decreased in the liver and increased in the serum of hepatic *Vps33b*-depleted mice compared to those in wild-type mice. Our study demonstrated that *Vps33b* influences the progress of liver metabolism both in bile acid circulation and lipid metabolism, which is involved in the progression of liver cholestasis in mice.

## Introduction

Arthrogryposis, renal dysfunction and cholestasis (ARC) syndrome (OMIM 208085), an autosomal recessive multiorgan disorder that was originally described in 1973 by Lutz-Richner typically presents with neurogenic arthrogryposis, multiplex congenita, renal tubular dysfunction and neonatal cholestasis with bile duct hypoplasia. This disorder mostly affects the offspring of consanguineous unions, where patients usually die within a few months of birth (Horslen et al., 1994). Until now, approximately 75% of ARC patients were diagnosed with a *VPS33B* (Vacuolar protein sorting-associated protein 33B) mutation (Cullinane et al., 2009), while others were characterized with a mutation in *VIPAR*, a VPS33B interacting protein, or, *Vps16B* (Ackermann et al., 2014; Rogerson and Gissen, 2018).

VPS33B, a 617 AA protein of the Sec-1/Muc18 (SM) family (Huizing et al., 2001), is involved in the vesicular intracellular trafficking process and protein interactions (Chen C.H. et al., 2017). SM proteins together with SNAREs are generally required for membrane fusion through regulating the formation of quaternary SNARE complexes, including GTPases and other tethering proteins containing long coiled domains. (Sudhof and Rothman, 2009; Lobingier and Merz, 2012). The general role of these proteins in membrane trafficking demonstrated the vital role of *Vps33b* in intracellular trafficking functions.

The function of VPS33B and its association with ARC disorder has been reported according to the many clinical features of this disease. For example, VPS33B is one of the first proteins found to be essential for a-granule biogenesis according to ARC syndrome. The absence of *Vps33b* causes a lack of a-granules, soluble cargo and p-selectin, a granule-specific membrane protein, in platelets leading to a bleeding diathesis, secondary to platelet dysfunction in ARC syndrome (Hayes et al., 2004; Dai et al., 2016). Second, *Vps33b* was reported to take participate in the maturation of phagosomes and endosomes following microbial antigen ingestion (Akbar et al., 2016). The lack of *Vps33b* in Drosophila contributes to accelerated inflammatory responses and microbial stimulation of pattern-recognition receptors such as toll-like receptors. Furthermore, severe liver histological change is one of the most important features of ARC syndrome, including cholestasis, bile duct hypoplasia and lipofuscin granule deposition (Eastham et al., 2001). Currently, although VPS33B function has been partly studied, there is no curative therapy for ARC syndrome, which is involved in multiple clinical features.

Clinical therapy for ARC simply relieves patient discomfort, such as ursodeoxycholate therapy, a promising drug for cholestasis that has been used to decrease pruritus mainly caused by the increased level of serum bilirubin. In that case, it suggests that bile acid metabolism disturbances might have a role in the etiopathogenesis of cholestasis in these ARC cases (Abdullah et al., 2000). Unfortunately, no further studies were conducted to reveal the role of *Vps33b* in the homeostasis of bile acids. Otherwise, it is reported that liver transplantation can significantly improve ARC patients’ clinical symptoms and prolong patient growth status (Dehghani et al., 2013). Therefore, dysregulated liver function is a severe sign among ARC patients who bear the *VPS33B* mutation. Thus, it is clear that VPS33B may play an important role in the liver, and mechanistic studies of VPS33B might be helpful to develop an ARC therapy or address other liver diseases.

Recently, the rise of omics studies, especially metabolomics and lipidomics, have become the preferred method to provide new insights into the etiology of liver diseases, new treatment modalities and a new understanding that may reveal potential therapeutic targets. For example, a metabolomics strategy has been used to map the bile acid profile *in vivo* and reveal how bile acid homeostasis is disrupted during the disease progression (Chen P. et al., 2017).

In the current study, hepatic *Vps33b*-depleted male mice were used to investigate the metabolomics and lipidomics profiles of hepatic *Vps33b* deficiency in order to map the bile acids deposition and lipid profile of *Vps33b* hepatic knockout mice. Furthermore, the role of *Vps33b* in bile acids homeostasis and lipid metabolism and the involved mechanisms were further studied to find out possible therapeutics targets of diagnostic markers.

## Materials and Methods

### Chemicals and Reagents

Serum biochemistry measurement kits (ALT, AST, ALP, and TBILI) were purchased from Shanghai Kehua Bio-Engineering Co., Ltd. (China). Serum TBA measurement kits were purchased from Nanjing Jiancheng Bioengineering Institute (China). All other solvents and regents were of analytical or HPLC grade when appropriate.

### Animal Handling

*Vps33b* hepatic knockout mice (*Vps33b^flox/flox^*, alb-cre) and wild-type mice (*Vps33b^flox/flox^*) with a C57BL/6 genetic background were obtained from Junling Liu’s laboratory (Shanghai Jiao Tong University School of Medicine, China). Mice were housed in stainless exhaust-ventilated closed-system cages in a specific-pathogen-free environment. Mice were maintained under a standard 12 h light/12 h dark cycle with water and a normal diet provided ad libitum. Animal experiments were performed in accordance with the guidelines of the Institutional Animal Care and Use Committee at Sun Yat-sen University (Guangzhou, China).

### Mice Generation and PCR Genotyping

The *Vps33b* floxed allele was generated as previous reported (Wang et al., 2018). Mice were bred by crossing *Vps33b^flox/flox^*, alb-cre and *Vps33b^flox/flox^* mice. A 1–2 mm portion of mouse tail was cut for genotyping at the age of 3 weeks. A one-step mouse genotyping kit was purchased from Vazyme Biotech Co., Ltd (China). The supernatant of tail lysis buffer was added into the PCR system with 2x Taq Plus Master Mix (Dye Plus), primers and DEPC water. The PCR procedure were performed by agarose gel electrophoresis. *Vps33b^flox/flox^*, alb-cre mice showed two bands at 300 and 606 bp, while *Vps33b^flox/flox^* mice only had only a 606 bp band. Primer sequence of genotyping are listed as follows: *Cre*-reverse primer *5′-ATTTGCCTGCATTACCGGTCG-3′* and *Cre*-forward primer *5′-CAGCATTGCTGTCACTTGGTC-3′; Vps33b*-floxed allele gene primer A1 *5′-CTGACTAGGGGAGGAGTAGAAGGT-3′*, A2 *5′-GTATCACTGAGTCACACACATCCA-3′* and A3 *5′-ATAGAGACGTTAGCAATTCGATCC-3′*.

### Sample Collection

*Vps33b^flox/flox^*, alb-cre mice were sacrificed at 3–4 months of age, and age-matched *Vps33b^flox/flox^* mice were used as wild-type controls. All procedures were undertaken with the approval of the Institutional Animal Care and Use Committee at Sun Yat-sen University (Guangzhou, China). Serum was obtained after centrifugation of the blood at 3000 rpm for 10 min at RT. Bile was transferred into a 1.5 mL Eppendorf tube and weighed. Livers, intestines and feces were collected for further study respectively.

### Serum Biochemistry

Serum activities of ALT, AST, ALP, TBILI, and TBA were measured by commercially available kits on an automatic biochemical analyzer.

### Liquid Chromatography/Mass Spectrometry (LC/MS) and Metabolomic Analysis

Samples preparation for metabolomic analysis were performed according to our previously reported methods with some slight modifications (Chen P. et al., 2017; Zeng et al., 2017; Zhang et al., 2017b). Ten microliters serum and five microliters bile were mixed with 67% aqueous acetonitrile in distilled water to remove the protein, followed by centrifugation at 18000 *g* for 20 min at 4°C to obtain the supernatant. Liver and intestinal tissues were homogenized with 50% aqueous acetonitrile in distilled water and centrifuged at 18000 *g* for 20 min at 4°C to precipitate the protein. Twenty microliters of feces-PBS homogenate (20 μg per 400 μL) were vortexed with 67% ACN, followed by centrifugation to obtain the supernatant.

The obtained supernatant was transferred to an UPLC vial. Five microliter aliquots of metabolic samples were injected into the UPLC-ESI-Q Exactive interfered system (Dionex Corporation, Sunnyvale, CA, United States, Thermo Fisher Scientific, Waltham, MA, United States). ACQUITY UPLC BEH C18 column 1.7 μm (2.1^∗^50 mm, Waters Corporation, Milford, MA, United States) was used to performed chromatography separation. Colum temperature was 60°C. The mobile phase consists of solvent A (0.1% formic acid in water) and solvent B (0.1% formic acid in acetonitrile) with the flow rate of 0.5 mL/min. The gradient program was as follows: 0 min 5% B, 0.5 min 5% B, 1 min 35% B, 6 min 60% B, 8 min 95% B, 9 min 95% B, 10 min 5% B, 10.5 min 5% B.

Electrospray negative ionization mode was used for analysis. The spray voltage was set to 3.5 kV. Capillary and auxiliary gas heater temperatures were set at 325 and 350°C respectively. Nitrogen was used as both the sheath gas at a flow rate of 60 arbitrary units and the auxiliary gas at a flow rate of 20 arbitrary units. Target SIM method which included the accurate Q/Z of bile acids was used to get higher sensitivity. The mass spectral data were aligned using SIEVE 2.2 (Thermo Fisher Scientific, Waltham, MA, United States). Then the extracted component was prepared for further analysis.

Multivariate data analysis was performed using SIMCA 13.0 software (Umetrics, Kinnelon, NJ, United States). Principal components analysis (PCA) and supervised orthogonal partial least squares discriminate analysis (OPLS-DA) models were used to analyze the data of tissues samples, such as the serum, liver, bile, intestine, and feces. Further identification of bile acids was conducted by comparing the retention time and fragmentation patterns with authentic standards according to our previously reported method (Chen P. et al., 2017).

### Liquid Chromatography/Mass Spectrometry (LC/MS) and Lipidomic Analysis

Methyl tert butyl ether (MTBE) method (Matyash et al., 2008) was used to extract lipids from the serum and liver tissues. Twenty microliters serum was vortexed by adding prechill methanol, MTBE, and ultrapure water successively for 30 s respectively. Then the mixture was centrifuged at 3000 × rpm for 10 min. The supernatant was obtained for vacuum drying. Twenty micrograms liver tissues were homogenized with PBS and lipids were extracted as we mentioned before (Li et al., 2018). Before injection, samples were re-suspended in 200 μL mixture of methanol/isopropanol (1:1, v/v) and centrifuged at 18,000 × *g* for 5 min at 4°C.

Chromatography separation was performed using an Ascentis Express C18 2.7 μm column (100 mm × 2.1 mm, Sigma-Aldrich, St. Louis, MO, United States) on a Thermo Scientific Dionex Ultimate 3000 UPLC-ESI-Q Exactive system. The chromatograpy conditions, including the consistent of mobile phase and linear gradient, were similar with our previous report (Li et al., 2018). Mass spectrometry was performed with electrospray positive (ESI^+^) and negative (ESI^-^) ionization modes. The main parameters for MS/MS included the following parameters: AGC target 1e^5^, maximum IT 65 ms, isolation window 1.2 m/z, normalized collision energy 25, 35 eV in positive mode, 20, 30, and 40 eV in negative mode, apex trigger 5–10 s, and dynamic exclusion 10.0 s. Ionization conditions were operated at a spray voltage of 3.5 kV and a capillary temperature of 300°C.

Lipidomic data processing was performed according to our previous report (Zhang et al., 2017a) using Lipid Search software (Thermo Scientific, San Jose, CA, United States).

### RNA Isolation and qRT-PCR Analysis

RNA isolation and qRT-PCR analysis of hepatic gene mRNA expression level was performed as described previously (Chen P. et al., 2017). The primer sequences were obtained from Primer Bank and synthesized by Thermo Fisher Scientific. Primer sequences were listed in Supplementary Table 1.

### Total Protein Extraction and Western Blot Analysis

Liver total protein extraction and western blot analysis were performed as described previously. Bolts were incubated with primary antibody against BSEP (F-6) (Santa Cruz Biotechnology, Santa Cruz, CA, United States), E-CADHERIN1 (Gentex Corporation, Zeeland) and GAPDH (Cell Signaling Technologies, Danvers, MA, United States).

### Histological Analysis

Liver specimens were fixed in 10% formalin solution and processed routinely for paraffin embedding. Sections (4 μm thick) were deparaffinized and then stained with hematoxylin and eosin solutions (H&E) and examined under a light microscope (Nikon 80i, Japan). Immunohistological staining was performed with a primary antibody against CLAUDIN-1 (Abcam, San Francisco, CA, United States).

### Statistical Analysis

Each group was consisted of six animals. All values are expressed as the means ± SD. Statistical analysis was performed by unpaired Students’ *t*-test or Mann–Whitney *U*-test with Prism 6 (GraphPad Software Inc., San Diego, CA, United States) or SPSS statistical software. A *p*-value of less than 0.05 was considered statistically significant.

## Results

### Hepatic *Vps33b* Depletion Induced Minor Cholestastic Liver Injury

Compared to the age-matched male wild-type mice, hepatic *Vps33b*-depleted mice fed a controlled diet were identified for *Vps33b* depletion efficiency in the liver. *Vps33b* mRNA and protein levels were significantly decreased in *Vps33b^flox/flox^*, alb-cre mice compared with those in the *Vps33b^flox/flox^* mice (Supplementary Figures 1A–C).

**FIGURE 1:**
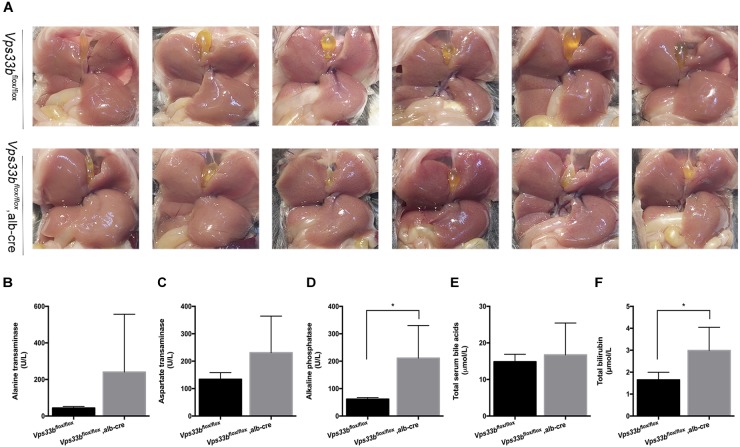
*Vps33b^flox/flox^*, alb-cre mice had distinctly smaller gallbladders **(A)** and higher serum biochemistry levels **(B–F)** compared to those of *Vps33b^flox/flox^* mice. **(A)** *In situ* view of gallbladders from the two groups; **(B)** ALT, **(C)** AST, **(D)** ALP, **(E)** total bile acids, and **(F)** total bilirubin levels in serum of adult male mice (3–4 months of age) with the indicated genotypes. Values are expressed as the mean ± standard deviation (*n* = 5 per group). ^∗^*p* < 0.05 vs. *Vps33b^flox/flox^* mice.

**FIGURE 2:**
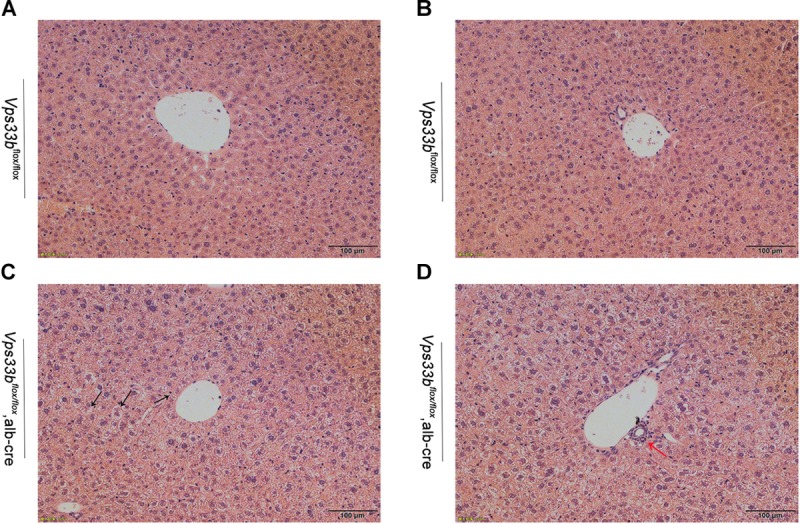
Representative H&E-stained livers from *Vps33b^flox/flox^* **(A,B)** and *Vps33b^flox/flox^*, alb-cre mice **(C,D)**. Necrosis of hepatocytes (black arrow) and ductal proliferation (red arrow) were observed.

Furthermore, no significant differences in body weight and liver/body weight ratio were observed between *Vps33b^flox/flox^* and *Vps33b^flox/flox^*, alb-cre mice (Supplementary Figures 2A,B). Although no significant differences in bile volume were observed between *Vps33b^flox/flox^* and *Vps33b^flox/flox^*, alb-cre mice, *Vps33b^flox/flox^*, alb-cre mice displayed a distinctly smaller gall bladder. In addition, serum biochemistry analysis showed that ALP and serum total bilirubin levels in *Vps33b^flox/flox^*, alb-cre mice were higher than those in *Vps33b^flox/flox^* mice (Figure 1E,F). Liver damage indexes, such as serum ALT and AST levels, were also elevated in *Vps33b^flox/flox^*, alb-cre mice compared with those in *Vps33b^flox/flox^* mice (Figure 1). In addition, histological analysis showed hepatocyte degeneration, necrosis and ductal proliferation around the portal area in *Vps33b^flox/flox^*, alb-cre mice (Figure 2). These results indicated that hepatic *Vps33b* knockout mice displayed cholestasis and slight liver injury compared to *Vps33b^flox/flox^* mice.

### Hepatic *Vps33b* Depletion Disrupted Bile Acid Homeostasis in Mice

**FIGURE 3:**
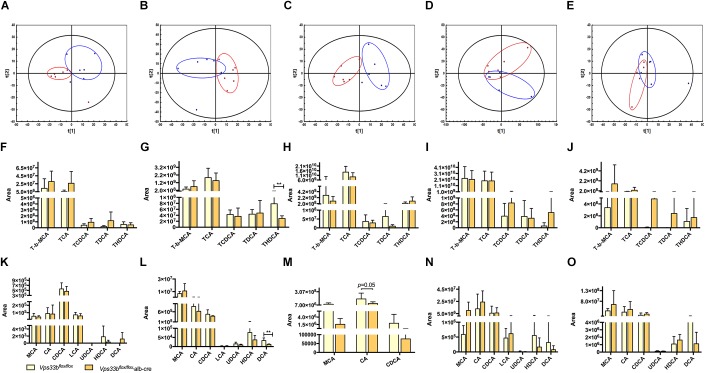
Principal component analysis (PCA) score plot **(A–E)** of *Vps33b^flox/flox^* mice (Blue) and *Vps33b^flox/flox^*, alb-cre mice (Red). ESI^-^negative mode; Serum **(A)**; Liver **(B)**, Bile **(C)**; Intestine **(D)** and Feces **(E)**. Bile acid components in serum **(F,K)**, liver **(G,L)**, bile **(H,M)**, intestine **(I,N)** and feces **(J,O)** of mice. Conjugated bile acids **(F–J)**; Unconjugated bile acids **(K–O)**.

Metabolomics analysis was performed to examine dynamic changes of endogenous metabolites in the serum, liver, bile, intestine and feces. Unsupervised PCA was used to analyze the data sets from *Vps33b^flox/flox^* mice and *Vps33b^flox/flox^*, alb-cre mice. Each point represented a mouse in their group. Significant separation of serum, liver and bile samples was observed in the PCA model, as shown by the distribution of *Vps33b^flox/flox^* and *Vps33b^flox/flox^* alb-cre mice in different quadrants, indicating a significant difference in endogenous metabolomes between the two groups of mice (Figure 3A–C). However, both the intestine and feces maintained a cross-distribution of metabolites in the PCA score plot (Figure 3D,E). These results were in accordance with the trend observed in the serum biochemistry. We further performed targeted metabolomics analysis of bile acids to gain a full understanding of how hepatic *Vps33b* depletion influences bile acid homeostasis. The extracted ions from mass spectrum of mice samples were confirmed by comparing with authentic bile acids standard according to retention time and MS/MS (Supplementary Figure 3). Bile acids were classified as conjugated and unconjugated types, and their relative amount in the tissues of mice was calculated (Figure 3F–O).

TCA and T-beta MCA consisted of a majority of conjugated bile acids in mouse tissue samples. Both were elevated in serum and reduced in bile by hepatic depletion of *Vps33b*. T-beta MCA was increased in the livers of *Vps33b^flox/flox^*, alb-cre mice, while TCA was prone to reduction compared with those in the *Vps33b^flox/flox^* mice. These major conjugated bile acid levels in the intestine were not changed by *Vps33b* deficiency in the liver. Overall, combined with other conjugated bile acids, such as TCDCA, TDCA and THDCA, conjugated bile acids were mostly increased in the serum of *Vps33b^flox/flox^*, alb-cre mice, while decreased in the liver and bile compared with those in the *Vps33b^flox/flox^* mice. However, unconjugated bile acids were generally reduced in the serum, liver and bile of *Vps33b^flox/flox^*, alb-cre mice. Furthermore, hepatic *Vps33b* depletion seemed to have no effect on bile acid distribution between the intestine and feces (Supplementary Figure 4). In summary, the bile acid distribution pattern was clearly changed in hepatic *Vps33b*-depleted mice compared with that in the *Vps33b^flox/flox^* mice, demonstrating that *Vps33b* plays an important role in sustaining bile acid homeostasis.

### Regulation of Hepatic Bile Acid Homeostasis-Related Gene Expression and Disruption of Hepatocyte Polarity Caused by *Vps33b* Deficiency

**FIGURE 4:**
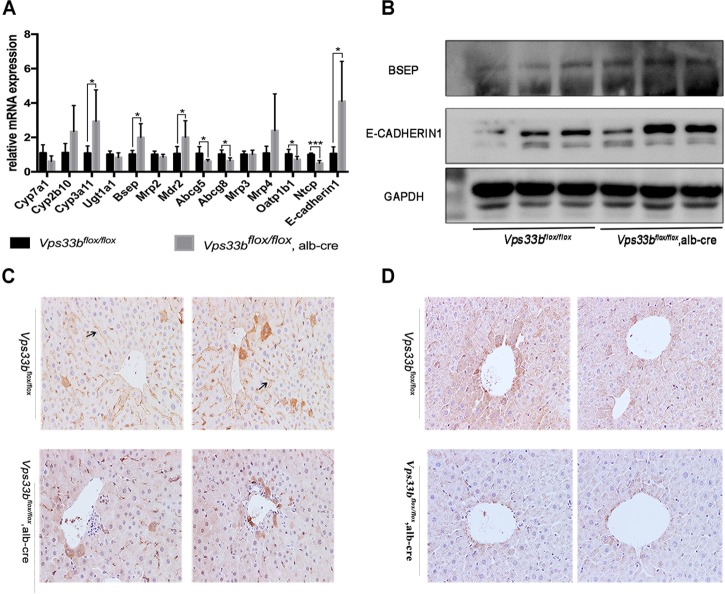
Bile homeostasis-related gene expression levels **(A)**. Western blotting **(B)** and IHC **(C)** detection of BSEP, and *E*-cadherin1 **(A,B)** and CLAUDIN-1 **(D)** cell junction protein expression levels.

According to the disrupted bile acid metabolomics pattern in *Vps33b^flox/flox^*, alb-cre mice, we detected several bile acid homeostasis-related gene expression levels in the liver (Figure 4). *Cyp7a1* converts cholesterol to 7a-hydroxycheolesterol, which is the rate-limiting enzyme of bile acid formation in the liver. The mRNA expression level of *Cyp7a1* was downregulated in *Vps33b^flox/flox^*, alb-cre mice compared to that in *Vps33b^flox/flox^* mice. However, Cyp450s enzymes, such as *Cyp2b10* and *Cyp3a11*, which function in the formation of hydrophilic bile acids, were significantly upregulated in the *Vps33b^flox/flox^*, alb-cre mice.

In addition, mRNA expression of hepatic canalicular membrane transporters (*Bsep* and *Mdr2*) were upregulated in *Vps33b^flox/flox^*, alb-cre mice, while *Abcg5/8* expression was downregulated compared to those in *Vps33b^flox/flox^* mice. Another canalicular transporter, *Mrp2*, was unchanged in *Vps33b^flox/flox^*, alb-cre mice. In addition, sinusoidal membrane multidrug resistance protein bile acid efflux transporters (*Mrp3* and *Mrp4*) were not significantly regulated by hepatic *Vps33b* depletion. However, bile acid reuptake transporters (*Oatp1b1* and *Ntcp*) were significantly downregulated in *Vps33b^flox/flox^*, alb-cre mice (Figure 4A).

*Vps33b* function was related to hepatocyte polarity, which is vital for cell junction formation and specific protein localization. Furthermore, we performed immunohistochemistry staining of the bile acid export pump BSEP and measured the levels of representative cell junction proteins (E-CADHERIN1, CLAUDIN-1). As shown in Figure 4A,B, BSEP mRNA and protein levels were significantly increased in *Vps33b^flox/flox^*, alb-cre mice compared to those in *Vps33b^flox/flox^* mice. Moreover, BSEP distribution features were subdued in *Vps33b^flox/flox^*, alb-cre mice, which were no longer localized along the specific side of hepatocytes. Livers express different kinds of cell tight junction proteins, and we chose E-CADHERIN1 and CLAUDIN-1 for detection. Increased E-CADHERIN1 levels and decreased CLAUDIN-1 levels were observed in *Vps33b^flox/flox^*, alb-cre mice compared with those in the *Vps33b^flox/flox^* mice (Figure 4B,D). Collectively, these data indicated that *Vps33b^flox/flox^*, alb-cre mice displayed disrupted bile acid homeostasis in accordance with an altered targeted-bile acid metabolomics pattern compared to that in *Vps33b^flox/flox^* mice. Irregular BSEP localization and altered cell tight junction expression suggested impaired hepatocyte polarity caused by *Vps33b* hepatic depletion.

### Altered Lipid Profiles in the Serum and Livers in *Vps33b*^flox/flox^, alb-cre Mice Indicated an Important Role of *Vps33b* in Lipid Metabolism

Because lipid metabolism is integrally connected to bile acid metabolism, we speculated whether the lipid metabolite pattern was also affected by hepatic *Vps33b* deletion. Upon UPLC-ESI-HRMS-based lipidomics analysis, we measured serum and liver samples of *Vps33b^flox/flox^*, alb-cre mice and *Vps33b^flox/flox^* mice to reveal the lipid profiles within specific lipid species.

**FIGURE 5:**
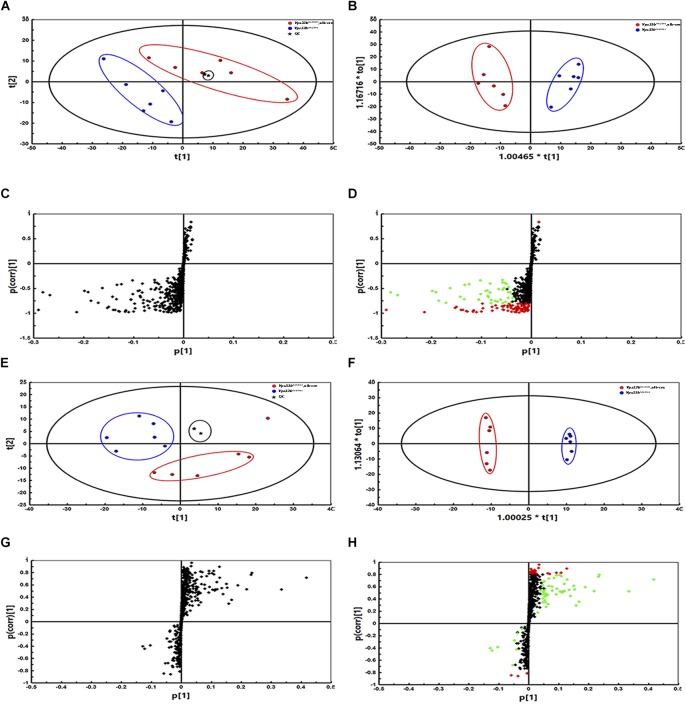
Principal component analysis, OPLS-DA score plot and *s*-plot of serum and liver lipid profiles in *Vps33b^flox/flox^*, alb-cre mice and *Vps33b^flox/flox^* mice. The PCA as well as OPLS-DA of serum **(A,B)** and liver **(E,F)** show a clear difference between the two groups (blue dots for *Vps33b^flox/flox^* mice, red dots for *Vps33b^flox/flox^*, alb-cre mice and black dots for QC control). The supervised multivariate analysis shows R2Y = 0.94 and Q2 = 0.877 in serum, while R2Y = 0.99 and Q2 = 0.498 in livers. *s*-plots **(C,D,G,H)** highlighted the possible calculated lipid components with *p*-value less than 0.05. [green dots represent VIP > 0.8, while red dots represent *p*(corr) < -0.6 or *p*(corr) > 0.6].

**FIGURE 6:**
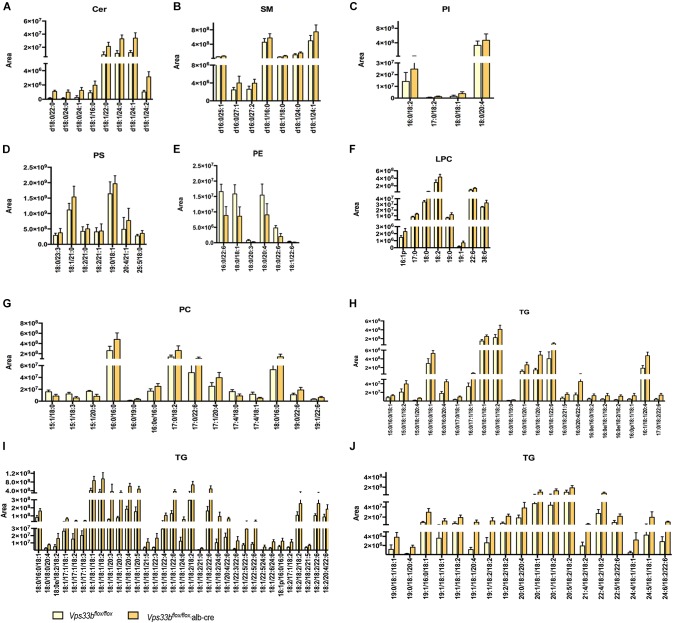
Lipid catalogs and species in serum were significantly different between *Vps33b^flox/flox^*, alb-cre and *Vps33b^flox/flox^*, mice. [**(A)** ceramides, **(B)** SM: sphingomyelin, **(C)** PI: phosphatidylinositol, **(D)** PS: phosphatidylserine, **(E)** PE: phosphatidyl ethanolamine, **(F)** LPC: lysophosphatidylcholine, **(G)** PC: phosphatidylcholine, and **(H–J)** TG: triglyceride].

**FIGURE 7:**
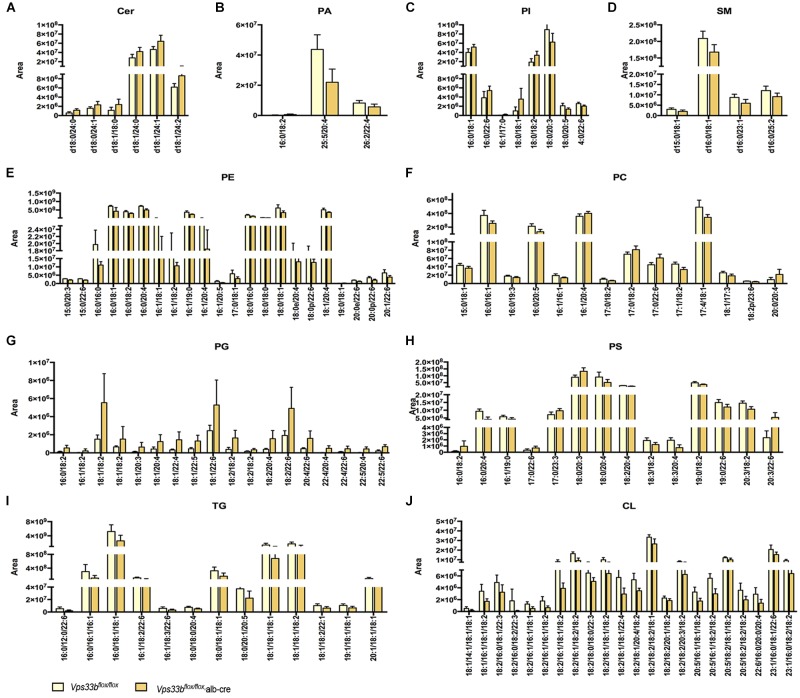
Lipid catalogs and species in the liver were significantly different between *Vps33b^flox/flox^*, alb-cre mice and *Vps33b^flox/flox^* mice. [**(A)** ceramides, **(B)** PA: phosphatidic acid, **(C)** PI: phosphatidylinositol, **(D)** SM: sphingomyelin, **(E)** PE: phosphatidyl ethanolamine, **(F)** PC: phosphatidylcholine, **(G)** PG: phosphatidyl glycerol, **(H)** PS: phosphatidylserine, **(I)** TG: triglyceride, **(J)** CL: cardiolipin].

Principal component analysis of serum and livers showed a clear difference between *Vps33b^flox/flox^* and *Vps33b^flox/flox^*, alb-cre mice (Figure 5A,E). Liver scatter plots for *Vps33b^flox/flox^* mice were in the second quadrants, while scatter plots for *Vps33b^flox/flox^*, alb-cre mice were mainly located in the third and fourth quadrants (Figure 5E). To identify the lipids that contribute to the unambiguous separation between *Vps33b^flox/flox^*, alb-cre mice and *Vps33b^flox/flox^* mice, Lipid Search software was used to identify lipid molecular species based on the accurate mass values of each ion and MS2 pattern. The outstanding matched mass spectrums were shown in Supplementary Figure 5. An OPLS-DA score plot (Figure 5B,F) and *s*-plot (Figure 5C,G) was performed to screen the specific lipids altered in *Vps33b^flox/flox^*, alb-cre mice among all identified lipid molecular species. VIP values > 0.8 and absolute *p*(corr) value > 0.6 are highlighted in green and red, respectively, in the *s*-plot (Figure 5D,H). Using the FDR (false discovery rate) test, screened specific lipid species of serum and livers with *p*-values < 0.05 are shown in the bar graph (Figure 6, 7). It is notable that many lipid species were altered in the serum and liver of hepatic *Vps33b*-depleted mice according to normalized heatmap pictures (Supplementary Figures 6, 7). TGs were the most abundant lipid species that were altered significantly in serum. Interestingly, regardless of the number of carbons or double bonds in the fatty acid chain, all species of TGs were significantly increased in serum but decreased in the liver of *Vps33b^flox/flox^*, alb-cre mice compared with those in the *Vps33b^flox/flox^* mice (Figure 6H–J, 7I). SMs were another type of lipid that exhibited a similar altered trend as TGs, in which the number of SM lipids was apparently less than that of TGs (Figure 6B, 7D). In addition, hepatic *Vps33b* depletion induced an upregulation of ceramides and a downregulation of PE in both serum and liver compared with those of *Vps33b^flox/flox^* mice (Figure 6A,E, 7E). Lipids, such as PI, PS and LPC, were significantly increased in the serum of *Vps33b^flox/flox^*, alb-cre mice (Figure 6C,D,F). However, some lipids were significantly altered with a different variation trend in *Vps33b^flox/flox^*, alb-cre mice, such as PC in serum and PI, PC, and PS in livers (Figure 6G, 7C,F,H). Moreover, PA and CL were increased, and PG was decreased in the livers of *Vps33b^flox/flox^*, alb-cre mice, which were not significantly changed in serum compared with those in the *Vps33b^flox/flox^* mice.

**FIGURE 8:**
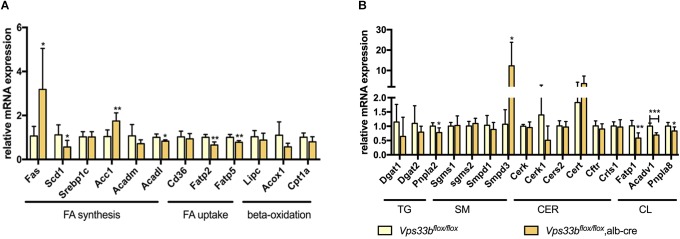
Lipid homeostasis related gene expression levels in the livers of *Vps33b^flox/flox^*, alb-cre mice and *Vps33b^flox/flox^* mice **(A,B)**. ^∗^*p*-value < 0.5, ^∗∗^*p*-value < 0.1, and ^∗∗∗^*p*-value < 0.001.

In addition, we detected the expression of lipogenesis-related genes and specific lipid metabolism-related genes. As shown in Figure 8, fatty acid uptake genes were significantly decreased in the livers of *Vps33b^flox/flox^*, alb-cre mice compared with those in the *Vps33b^flox/flox^* mice, while FA synthesis-related genes displayed an inconsistent change, as *Fas* and *Acc1* increased and *Scd1* decreased. TG metabolism-related genes were reduced, especially *Pnpla2*, in accordance with the decreased level of TG in the livers of *Vps33b^flox/flox^*, alb-cre mice. Meanwhile, CL-related genes were decreased as their amount was also lower in the livers of *Vps33b^flox/flox^*, alb-cre mice compared with those in the *Vps33b^flox/flox^* mice. Lipidomics analysis indicated that hepatic *Vps33b* regulated the lipid pattern of serum and livers in mice. Without *Vps33b* expression in the liver, the lipid species were distinct from *Vps33b^flox/flox^* mice, which might contribute to the pathology of cholestasis.

## Discussion

ARC syndrome, caused by *VPS33B* mutations, is a disease involving in multiple organs with a variety of clinical features (Cullinane et al., 2009), and alternative diagnostic approaches should be suggested to replace organ biopsies as a first-line diagnostic test for most children who are suspected to have ARC syndrome (Gissen et al., 2006). Therefore, basic studies have been increasingly performed to determine the tissue-specific function of *Vps33b*. For example, it has been reported that *Vps33b* plays an important role in platelet a-granule formation, and megakaryocyte *Vps33b*-depleted mice were used as an investigation model (Dai et al., 2016). However, few studies have investigated the role of *Vps33b* in the liver, with the exception of one study, which determined that *Vps33b* maintains hepatocyte polarity by using hepatic *Vps33b*-deficient mice (Hanley et al., 2017). Cholestasis is one of the syndrome of ARC, however, the role of hepatic *Vps33b* deficiency in the bile acids homeostasis and lipid metabolism remains unclear.

In this study, we investigated bile acid deposition and mapped lipid profiles of hepatic *Vps33b*-depleted mice based on an omics study strategy. We found that hepatic *Vps33b* depletion caused cholestasis and slight liver injury in mice, which was also commonly observed in ARC patients as previously reported. A targeted bile acid study confirmed disrupted bile acid homeostasis associated with *Vps33b* deficiency in the liver, and genes involved in bile acid circulation were also transcriptionally regulated. The mis-location of the BSEP apical membrane transporter was evidence of the loss of hepatocyte polarity. Cell junction proteins regulate the injured cell barrier, which might contribute to deteriorating cholestasis (Anderson, 1996). In addition, alterations in lipidomics profiles and the expression of genes involved in lipogenesis revealed a potentially vital role of *Vps33b* in liver lipid metabolism. Overall, our study characterized metabolic and lipidomic alterations in hepatic *Vps33b*-depleted mice (Supplementary Figure 8), which provided insights on the function of *Vps33b* on bile acids and lipid homeostasis.

Hepatic *Vps33b* knockout mice displayed a higher serum ALP level compared to *Vps33b^flox/flox^* mice, which is a marker for liver disease, specifically cholestasis. Additionally, the elevated serum ALT/AST levels indicated slight liver injury, which is similar to clinical ARC syndrome caused by a *Vps33b* mutation. In the clinic, ARC patients have been characterized by increased bilirubin (Gissen et al., 2006) with normal or lower levels of GGT, mildly elevated ALT/AST levels and substantially increased ALP levels (Malaki et al., 2012; Ilhan et al., 2016). We found that hepatic *Vps33b*-depleted mice displayed a similar phenotype to ARC patients in accordance with a previous report (Hanley et al., 2017). Therefore, hepatic *Vps33b*-depleted mice are a valuable experimental model for studying the therapeutic role of *Vps33b* in ARC syndrome.

Cholestasis is a complicated disease with disrupted bile acid homeostasis that is directly regulated by the synthesis and disposition systems consisting of cytochrome P450 enzymes, bile acid transporters and tight junctions (Gissen and Arias, 2015). We found that several CYP450 enzymes and bile acid transporters were transcriptionally altered in the livers of *Vps33b^flox/flox^*, alb-cre mice compared to those in *Vps33b^flox/flox^* mice. There is a metabolism loop that stabilizes the bile acid pool. Targeted bile acid metabolomics analysis indicated elevated taurine-conjugated bile acids in serum, which was decreased in the liver and bile of *Vps33b^flox/flox^*, alb-cre mice compared with those in *Vps33b^flox/flox^* mice. Our results were consistent with previous studies. In a previous study, an increase in TCA levels was observed in the plasma of *Vps33b^flox/flox^*, alb-cre mice fed with 0.5% CA chow. Furthermore, it was reported that 0.5% CA-fed *Vps33b^flox/flox^*, alb-cre mice showed a slight decrease in TCA levels in bile, but this decrease was not statistically significant (Hanley et al., 2017). Overall, alterations of bile acids among different tissues characterized the pathogenic conditions of cholestasis in hepatic *Vps33b*-depleted mice. The self-protection function in the liver allows it to attenuate injury under pathogenic conditions, which was observed in our results as the downregulated expression of bile acid synthesis enzyme *Cyp7a1* and bile acid reuptake transporters *Oatp1b1* and *Ntcp*. In addition to *Bsep*, the expression of other bile acid export transporters, *Mrp2/3/4*, did not change.

A clinical study demonstrated that the *VPS33B* mutation in ARC patients induced an unusual distribution of canalicular plasma membrane proteins. Liver biopsies of ARC patients revealed a clear disturbance in carcinoembryonic antigen (CEA) localization, which was present only at the canaliculus (Gissen et al., 2004). Furthermore, some BSEPs seemed to be expressed only at the basolateral hepatocyte membrane in the patient’s liver, which is supposed to be sorted to the apical membrane via RAB11A-positive apical recycling endosomes (Cullinane et al., 2010) to export bile acids from hepatocytes to the bile duct. In addition, the localization of another apical membrane transporter of hepatocytes, MRP2, did not change. In our study, the BSEP expression pattern in hepatocytes of hepatic *Vps33b*-depleted mice was quite different from those of *Vps33b^flox/flox^* mice. As an apically localized protein, BSEP might be predominantly distributed in the cytoplasm in the intrahepatic ABC-transporter pool, which is in an inactive state but is trafficked to the apical membrane to function as a bile acid exporting pump (Baier et al., 2006). It is clear that liver BSEP in *Vps33b^flox/flox^* mice completed this intracellular trafficking process, which was stained around the apical membrane of hepatocytes and a small bile duct through its 3D structures (de Aguiar Vallim et al., 2013). However, *Vps33b^flox/flox^*, alb-cre mice showed more cytoplasmic staining without representative localization in membranes compared to *Vps33b^flox/flox^* mice, even though its expression level was elevated in the liver. Overall, we concluded that mislocalization of BSEP contributed to cholestasis caused by hepatic *Vps33b* deficiency.

Cell junction proteins play an important role in sustaining hepatocyte polarity, which may serve as a belt junction between hepatocytes to prevent bile acid invasion from the bile duct. A study of mIMCD-3 cell polarity clarified that *Vps33b* deficiency caused structural and functional abnormalities, such as downregulated CLAUDIN-1 and E-CADHERIN1 expression (Cullinane et al., 2010). The absence of CLAUDIN-1, a key regulator of paracellular permeability, leads to a severe autosomal recessive disorder whose phenotype is similar to ARC syndrome (Colegio et al., 2003; Hadj-Rabia et al., 2004). Together with a previous report, CLAUDIN-1 displayed irregular and tortuous distribution in the liver of hepatic *Vps33b* knockout mice (Hanley et al., 2017), indicating that downregulated expression of CLAUDIN-1 in *Vps33b^flox/flox^*, alb-cre mice resulted in abnormalities in hepatocyte polarity. Furthermore, we found a converse change in E-CADHERIN1 expression in *Vps33b^flox/flox^*, alb-cre mice, which was supposed to be reduced as characterized by impaired cell polarity. However, the involvement of *E-cadherin* in regulating liver pathophysiology remains unclear (Gonzalez-Sanchez et al., 2015). As we mentioned previously, *Vps33b* functions on the apical protein sorting procedure via RAB11A-positive apical recycling endosomes. One possible pathway by which E-CADHERIN1 reaches the cell surface involves the Rab11-positive recycling complex rather than direct movement from the Golgi complex to the plasma membrane (Lock and Stow, 2005). Therefore, the correct localization of E-CADHERIN1, rather than its expression, might also be critical for liver function.

Cholestatic liver disease disturbs lipid absorption and metabolism. Furthermore, biliary secretion of cholesterol and phospholipids via ABCG5/8 and MDR2 in mice plays an important role in lipid homeostasis (Hanley et al., 2017). A previous study reported the mislocalization of ABCG8 in hepatic *Vps33b* knockout mice. In our study, we found a significant alteration in the expression of these transporters in *Vps33b^flox/flox^*, alb-cre mice. Additionally, different serum and liver lipid profiles with altered lipid-related gene expression in the liver were observed in *Vps33b^flox/flox^*, alb-cre mice. Serum TG, which is one of the signatures of intrahepatic cholestasis of pregnancy and PFIC in the clinic (Jankowska et al., 2016), showed an increasing trend in *Vps33b^flox/flox^*, alb-cre mice, which is consistent with the reduction of mRNA expression of fatty acid uptake genes Fatp2 and Fatp5 and TG synthesis genes.

Sphingomyelins (SMs), which are hydrolyzed by sphingomyelin phosphodiesterases (SMPDs) to form ceramides, act as secondary messengers in many physiological processes including apoptosis. Generally, the SM level is controlled by the balance of SM synthase (SGMS) and SMPD function. SMPD inhibitors have been reported to protect against bile acid-induced primary hepatocyte apoptosis (Qiao et al., 2002). Additionally, LCA-induced intrahepatic cholestasis showed elevated levels of ceramides (Cer) in mouse livers and hepatic expression of *Smpd3*, which were indicated as key factors that accelerated cholestasis (Matsubara et al., 2011). As we have shown in this study, hepatic *Vps33b* knockout mice had a disturbed balance of Cer and SM levels in mouse livers, with a significant increase in Smpd3 expression in *Vps33b^flox/flox^*, alb-cre mice.

After mapping the lipid profiles in *Vps33b^flox/flox^*, alb-cre mice, we found that the lipid components of *Vps33b* hepatic knockout mice were altered significantly compared to those of *Vps33b^flox/flox^* mice, indicating an important role of *Vps33b* in lipid metabolism. Further study of the relationship between *Vps33b* and lipid metabolism at the molecular level should be performed.

In summary, we investigated the phenotype of hepatic *Vps33b* knockout mice, providing additional evidence of the effect of *Vps33b* on ARC like cholestastic liver injury. In addition, our study demonstrated that *Vps33b* influences the bile acid homeostasis and lipid metabolism in mice, which is involved in the progression of liver cholestasis, indicating potential therapeutic targets and diagnostic markers of ARC.

## Data Availability

All datasets generated for this study are included in the manuscript and/or the Supplementary Files.

## Author Contributions

KF contributed to the study execution and manuscript preparation. CW supervised the weaning and genotyping of *Vps33b* hepatic knockout mice. YG, SF, HZ, CL, and LG contributed to the sample extraction for metabolomics studies and data analysis. JS and YJ reviewed the manuscript. HB, MH, and JL supervised the study progress and data analysis, revised the manuscript, and approved the final version of this manuscript for submission.

## Conflict of Interest Statement

The authors declare that the research was conducted in the absence of any commercial or financial relationships that could be construed as a potential conflict of interest.
