# Supplementary material for: Metabolomics and Lipidomics Reveal the Effect of Hepatic Vps33b Deficiency on Bile Acids and Lipids Metabolism
Source: Front Pharmacol. 2019 Mar 22;10:276. doi: 10.3389/fphar.2019.00276 (PMC6439481; doi:10.3389/fphar.2019.00276)
Supplement: Supplementary file 1 [file Table_1.docx]

Supplementary Material

Metabolomics and lipidomics reveal the effect of hepatic *Vps33b* deficiency on bile acids and lipids metabolism

Kaili Fu^1^, Conghui Wang^2^, Yue Gao^1^, Shicheng Fan^1^, Huizhen Zhang^1^, Jiahong Sun^1^, Yiming Jiang^1^, Conghui Liu^1^, Lihuan Guan^1^, Junling Liu^2^*, Min Huang^1^ and Huichang Bi^1^*

*** Correspondence:**

Huichang Bi: [bihchang@mail.sysu.edu.cn](mailto:bihchang@mail.sysu.edu.cn); Junling Liu: liujl@shsmu.edu.cn

# Supplementary Figures and Tables

**Supplementary Figure 1.** Hepatic *Vps33b* depletion efficiency in *Vps33b^flox/flox^*, alb-cre mice compared to *Vps33b^flox/flox^* mice (A and B). Immunoblot of whole liver lysates from 3 representative mice for each depicted genotype. GAPDH served as a loading control. (C) Liver *Vps33b* mRNA expression levels in each group. *p****<0.001 *vs.* *Vps33b^flox/flox^* mice.

**Supplementary Figure 2.** Body mass (A) and liver ratio (B) of v *Vps33b^flox/flox^*, alb-cre mice compared to those of *Vps33b^flox/flox^* mice. The differences were not statistically significant, as indicated by a *p-*value>0.05.

**Supplementary Figure 3.** The chromatogram of bile acids standards under accurate charge-to-mass ratio selected. CDCA, DCA, HDCA and UDCA: q/z 391.2842; TCA and T-b-MCA:q/z 514.2833; TCDCA, TDCA and THDCA q/z 498.2833; LCA 375.2893.

**Supplementary Figure 4.** Heatmap of bile acid deposition in serum, livers, bile, intestines and feces of *Vps33b^flox/flox^*, alb-cre mice and *Vps33b^flox/flox^* mice. Data were normalized to [-1-1]; yellow indicates an increase, and blue indicates a decrease.

**Supplementary Figure 5.** The representative matched spectrogram of altered lipids from Lipid Search software. (A) CER(d18:1/24:1), (B) SM(d16:0/18:1), (C) PE (18:0/18:1), (D) PC(16:0/16:0), (E)PI(18:0/18:1), (F)PA(24:2/22:6), (G)PS(18:0/20:4), (H) LPC(16:0), (I) TG(16:0/16:0/16:0) and (J) CL (18:2/16:1/16:1/22:6).

**Supplementary Figure 6.** Heatmap of significantly altered lipids in the serum of *Vps33b^flox/flox^*, alb-cre and *Vps33b^flox/flox^* mice. Data were normalized to [-2-2]; red indicates an increase, and blue indicates a decrease.

**Supplementary Figure 7.** Heatmap of significantlt altered lipids in the liver of *Vps33b^flox/flox^*, alb-cre and *Vps33b^flox/flox^* mice. Data were normalized to [-2-2]; red indicates an increase, and blue indicates a decrease.

**Supplementary Figure 8.** Alteration of bile acids and lipids in hepatic *Vps33b* depletion mice.

**Supplementary Table 1.** Primer sequences

| Primer name | Forward sequence | Reverse sequence |
| --- | --- | --- |
| *Gapdh* | AGGTCGGTGTGAACGGATTTG | GGGGTCGTTGATGGCAACA |
| *Cyp7a1* | GAACCTCCTTTGGACAACGGG | GGAGTTTGTGATGAAGTGGACAT |
| *Cyp2b10* | TGCTGTCGTTGAGCCAACC | CCACTAAACATTGGGCTTCCT |
| *Cyp3a11* | GGATGAGATCGATGAGGCTCTG | CAGGTATTCCATCTCCATCACAGT |
| *Ugt1a1* | GCTTCTTCCGTACCTTCTGTTG | GCTGCTGAATAACTCCAAGCAT |
| *Bsep* | TCTGACTCAGTGATTCTTCGCA | CCCATAAACATCAGCCAGTTGT |
| *Mdr2* | CAGCGAGAAACGGAACAGCA | TCAGAGTATCGGAACAGTGTCA |
| *Abcg5* | AGGGCCTCACATCAACAGAG | GCTGACGCTGTAGGACACAT |
| *Abcg8* | CTGTGGAATGGGACTGTACTTC | TGTTGTCACTTTCCGAGGAGA |
| *Mrp2* | GTGTGGATTCCCTTGGGCTTT | CACAACGAACACCTGCTTGG |
| *Mrp3* | CTGGGTCCCCTGCATCTAC | GCCGTCTTGAGCCTGGATAAC |
| *Mrp4* | CATCGCGGTAACCGTCCTC | CCGCAGTTTTACTCCGCAG |
| *Oatp1b1* | GCACTGCGATGGATTCAGGAT | AGCTTTGGTCGGTGTAGCTTG |
| *Ntcp* | CAAACCTCAGAAGGACCAAACA | GTAGGAGGATTATTCCCGTTGTG |
| *E-caherin1* | CAGGTCTCCTCATGGCTTTGC | CTTCCGAAAAGAAGGCTGTCC |
| *Fas* | GGAGGTGGTGATAGCCGGTAT | TGGGTAATCCATAGAGCCCAG |
| *Scd1* | TTCTTGCGATACACTCTGGTGC | CGGGATTGAATGTTCTTGTCGT |
| *Srebp1c* | GCAGCCACCATCTAGCCTG | CAGCAGTGAGTCTGCCTTGAT |
| *Acc1* | GATGAACCATCTCCGTTGGC | GACCCAATTATGAATCGGGAGTG |
| *Acadm* | AGGGTTTAGTTTTGAGTTGACGG | CCCCGCTTTTGTCATATTCCG |
| *Acadl* | TCTTTTCCTCGGAGCATGACA | GACCTCTCTACTCACTTCTCCAG |
| *Cd36* | CGCTTTCTGCGTATCGTCTG | GATGCACGGGATCGTGTCT |
| *Fatp2* | TCCTCCAAGATGTGCGGTACT | TAGGTGAGCGTCTCGTCTCG |
| *Fatp5* | CTACGCTGGCTGCATATAGATG | CCACAAAGGTCTCTGGAGGAT |
| *Lipc* | ATGGGAAATCCCCTCCAAATCT | GTGCTGAGGTCTGAGACGA |
| *Acox1* | TAACTTCCTCACTCGAAGCCA | AGTTCCATGACCCATCTCTGTC |
| *Cpt1a* | CTCCGCCTGAGCCATGAAG | CACCAGTGATGATGCCATTCT |
| *Dgat1* | TCCGTCCAGGGTGGTAGTG | TGAACAAAGAATCTTGCAGACGA |
| *Dgat2* | GCGCTACTTCCGAGACTACTT | GGGCCTTATGCCAGGAAACT |
| *Pnpla2* | GGATGGCGGCATTTCAGACA | CAAAGGGTTGGGTTGGTTCAG |
| *Sgms1* | GAAGGAAGTGGTTTACTGGTCAC | GACTCGGTACAGTGGGGGT |
| *Sgms2* | GAGACAGCAAAACTTGAAGGTCA | CCCGTTGGATAAGGTCTTGGG |
| *Smpd3* | CCCTCATCTTCCCATGTTACTGG | GGCGCTTCTCATAGGTGGTG |
| *Cerk* | CGGTACTGGTGTCGGAGATCA | GTGAATGCGAACGGATTTTCC |
| *Cerk1* | ACTGCTGGGCATAACGCTTTT | GAGGAGGATCTTGAGAGCCTT |
| *Cert* | AGTGCCTCTGACGATGTTCAC | ACCAGTTGCCAATTTGCATCA |
| *Cftr* | CTGGACCACACCAATTTTGAGG | GCGTGGATAAGCTGGGGAT |
| *Crls1* | GCCAGCTCGTATGAAAATCCA | GCAAAAACACCTAGTGCAACATT |
| *Fatp1* | CGCTTTCTGCGTATCGTCTG | GATGCACGGGATCGTGTCT |
| *Acadvl* | CTACTGTGCTTCAGGGACAAC | CAAAGGACTTCGATTCTGCCC |
| *Pnpla8* | GCAAGAAGTCTTTGTGGGAAACA | CTCACTTTTGTAAGTCCCTTGGG |
