# Supplementary figures and images for: Metabolomics and Lipidomics Reveal the Effect of Hepatic Vps33b Deficiency on Bile Acids and Lipids Metabolism
Source: Front Pharmacol. 2019 Mar 22;10:276. doi: 10.3389/fphar.2019.00276 (PMC6439481; doi:10.3389/fphar.2019.00276)

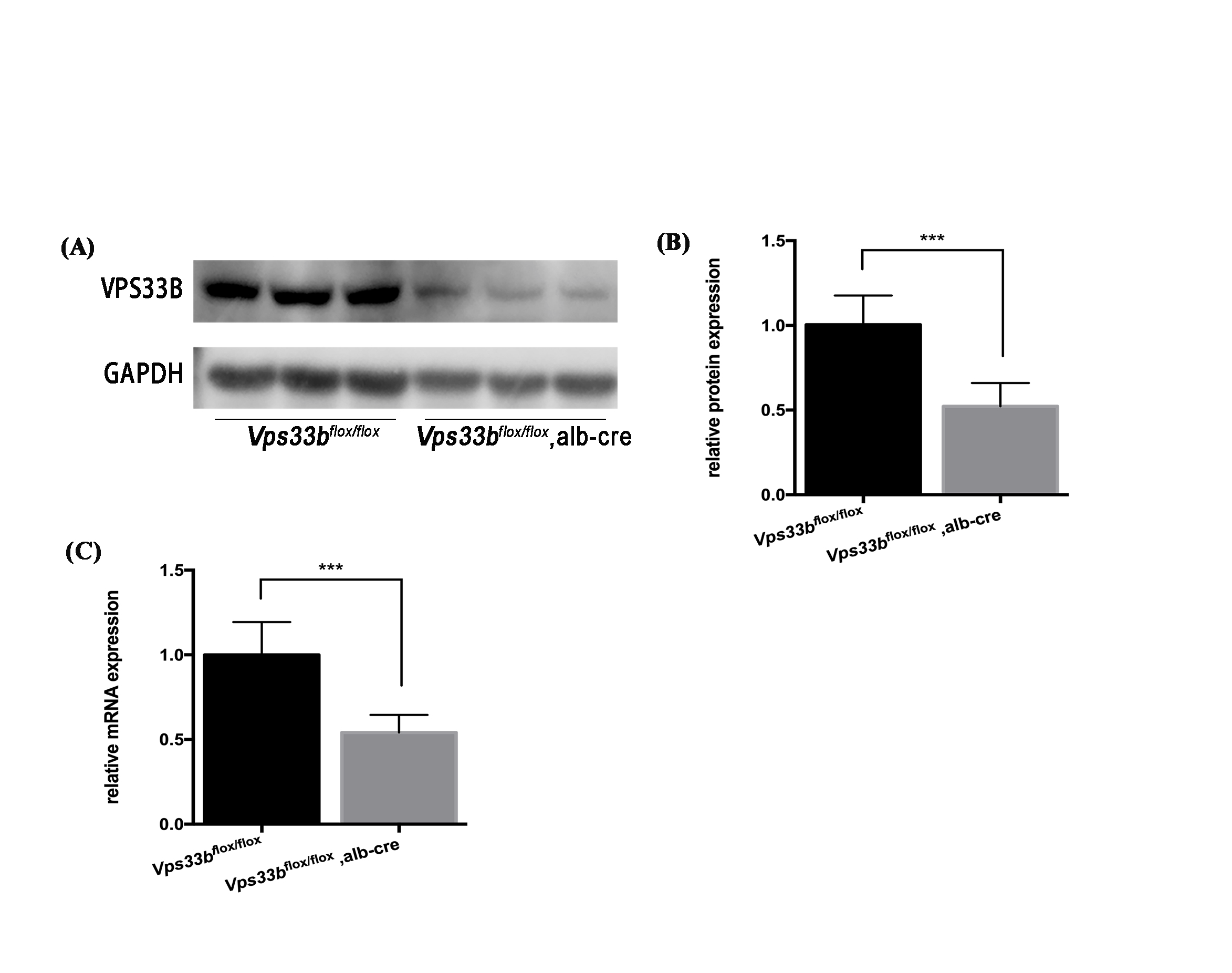

Supplement: Supplementary file 2 [file Image_1.TIFF]

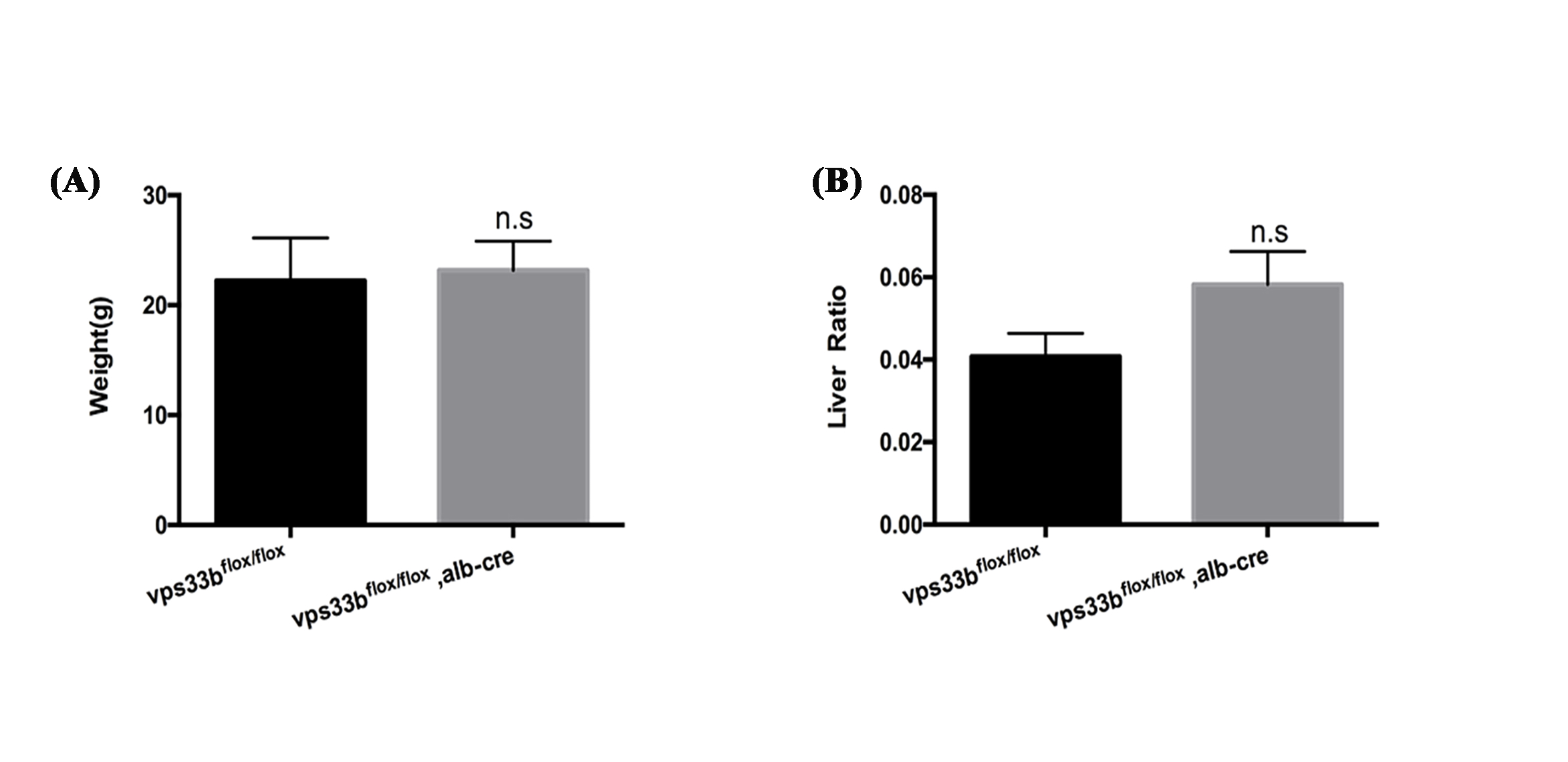

Supplement: Supplementary file 3 [file Image_2.TIF]

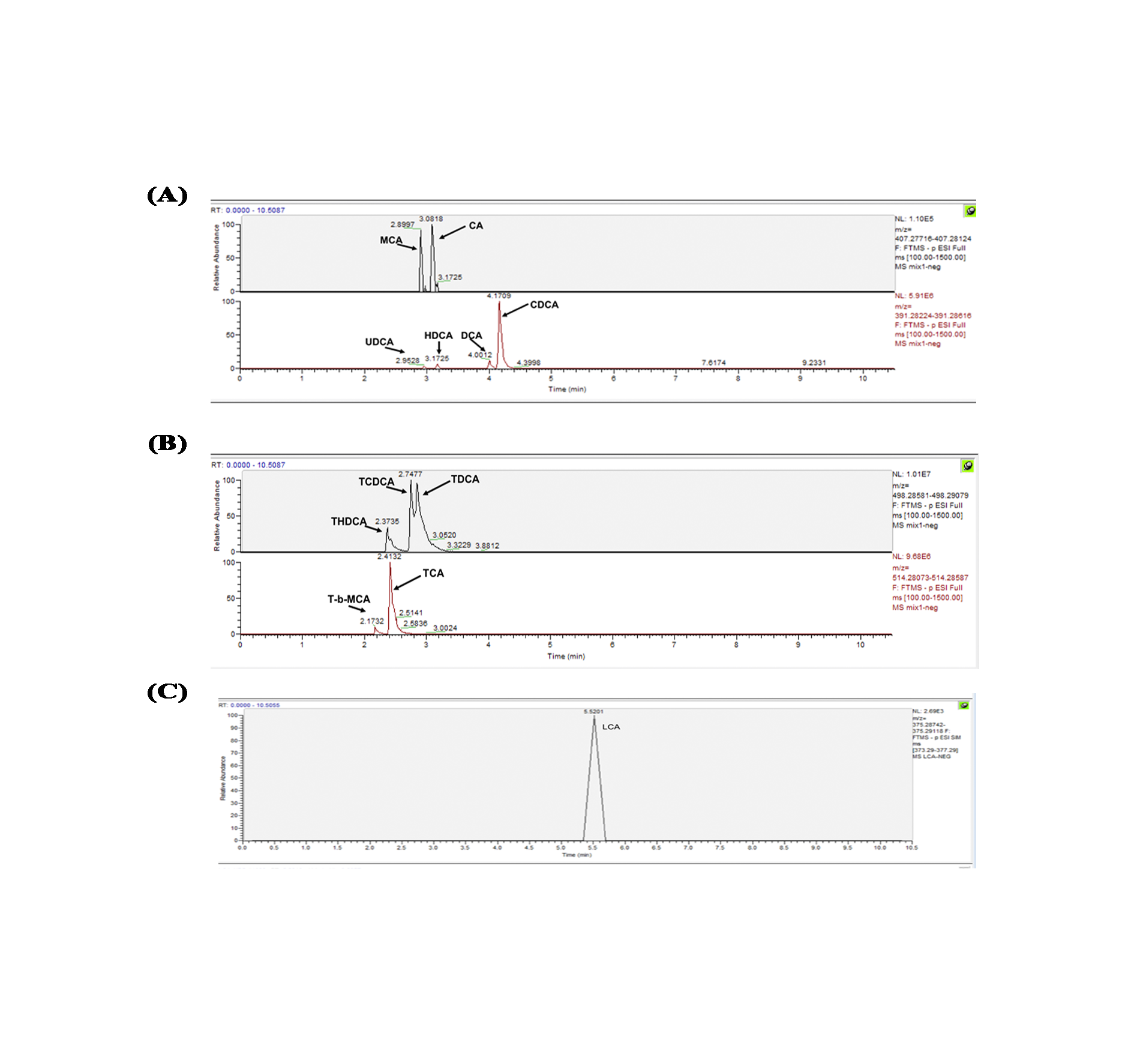

Supplement: Supplementary file 4 [file Image_3.TIF]

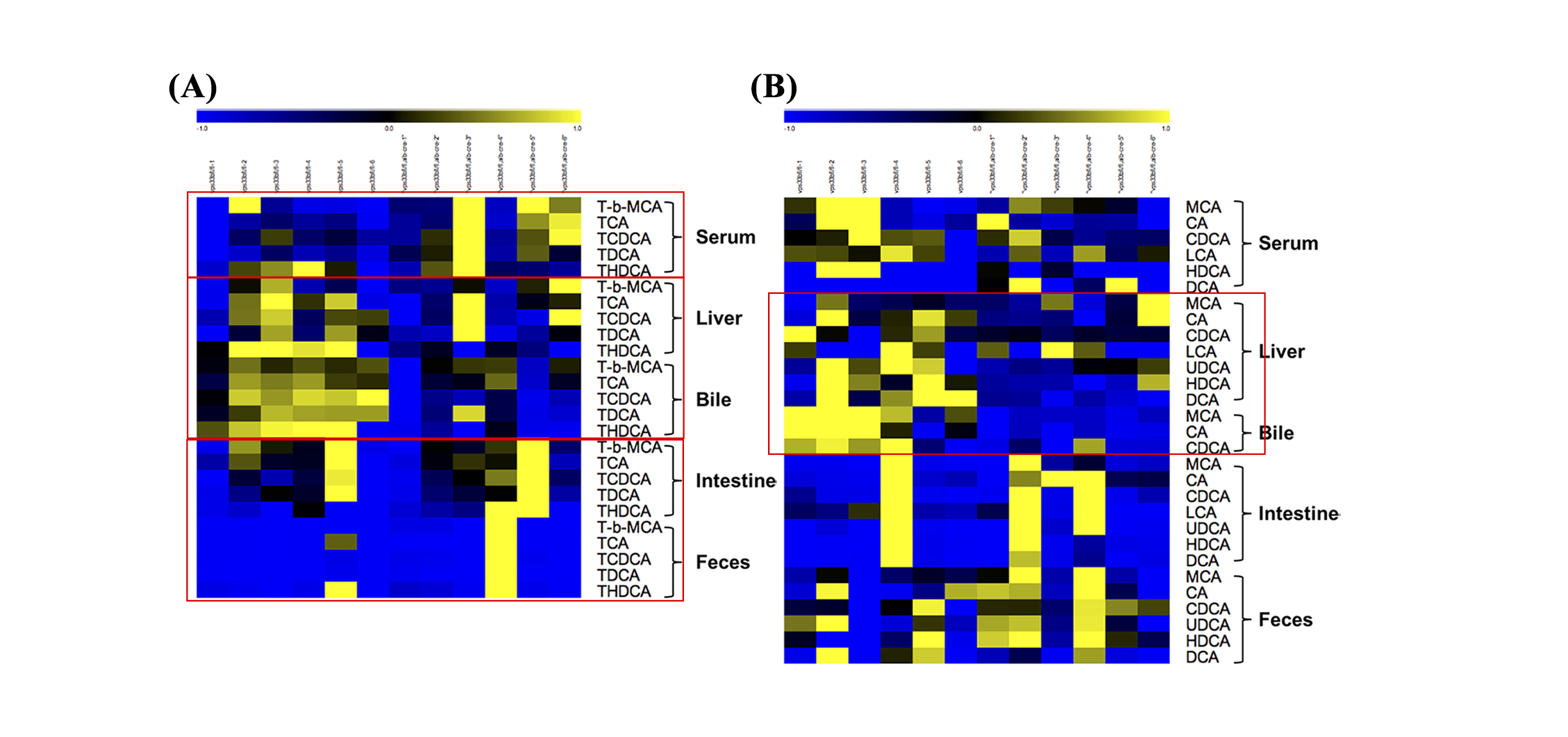

Supplement: Supplementary file 5 [file Image_4.TIFF]

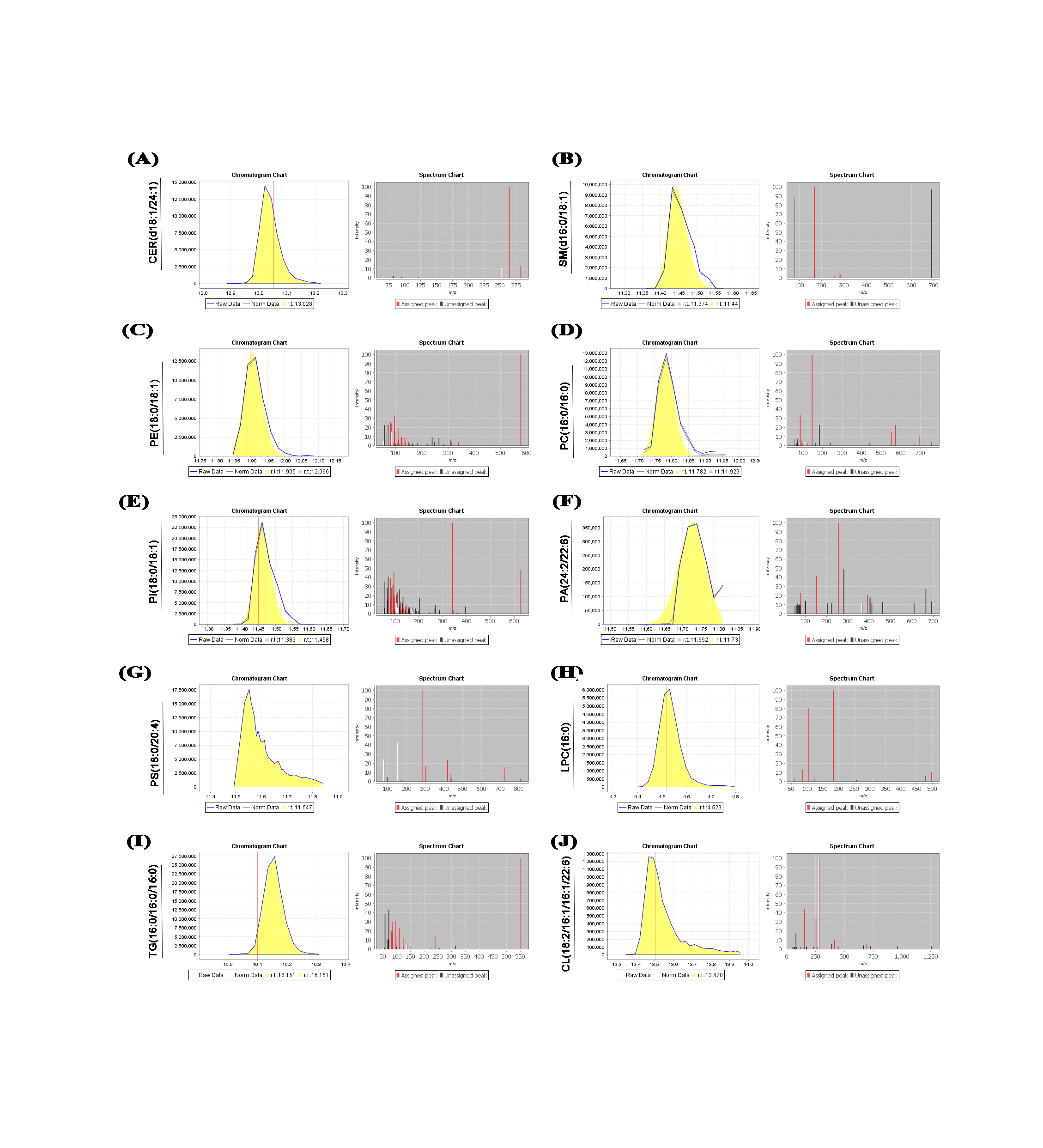

Supplement: Supplementary file 6 [file Image_5.TIF]

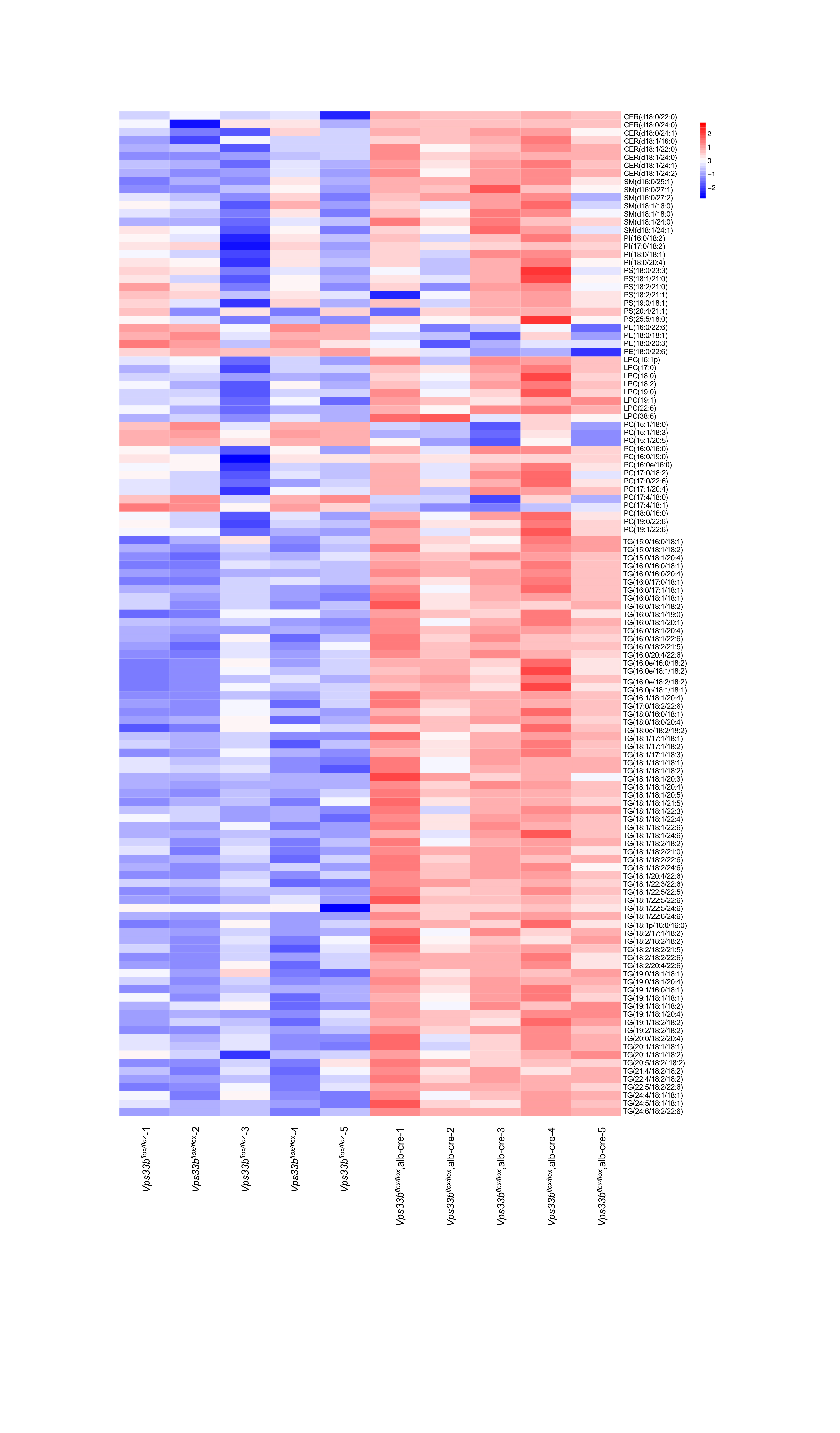

Supplement: Supplementary file 7 [file Image_6.JPEG]

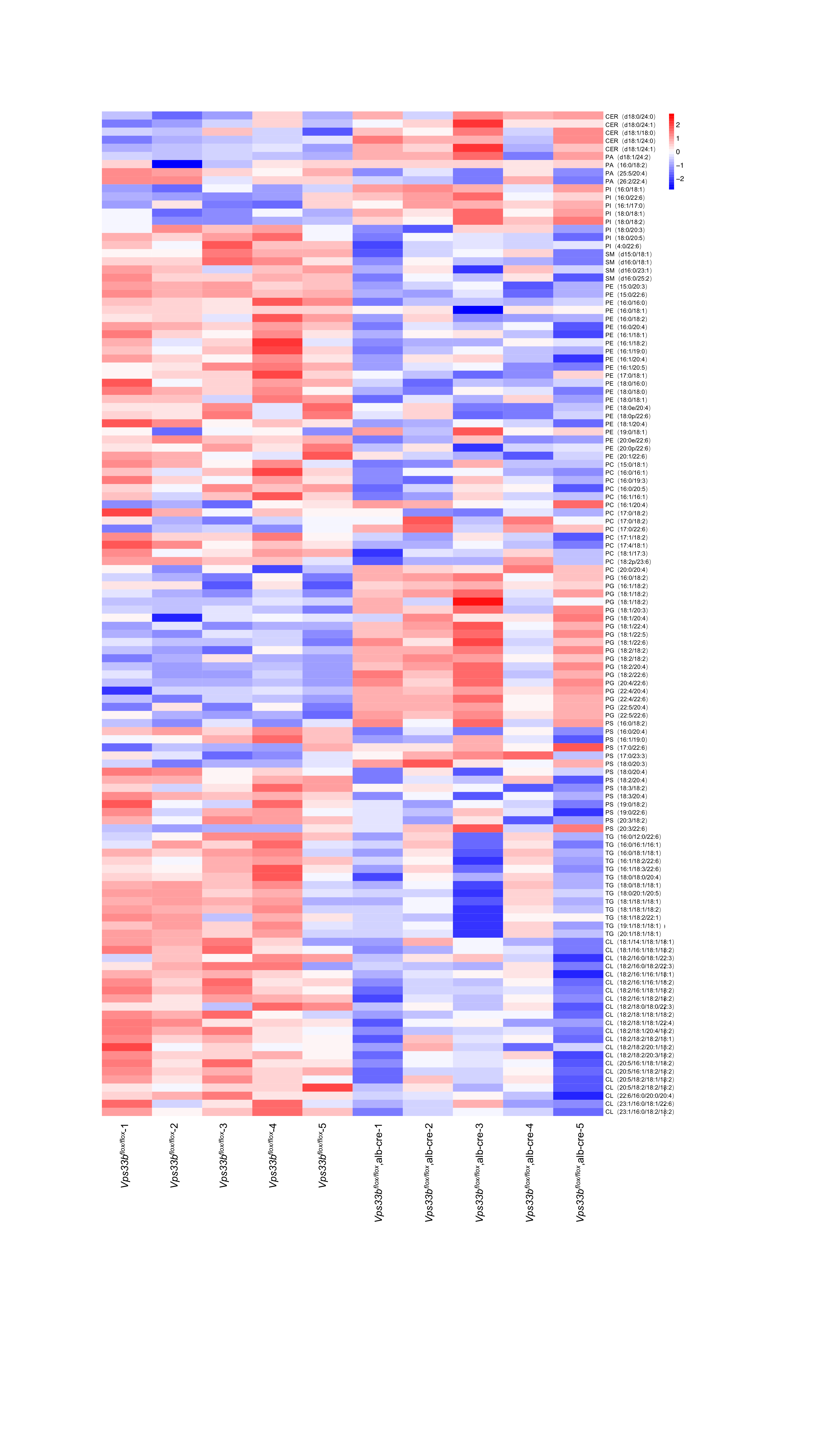

Supplement: Supplementary file 8 [file Image_7.JPEG]

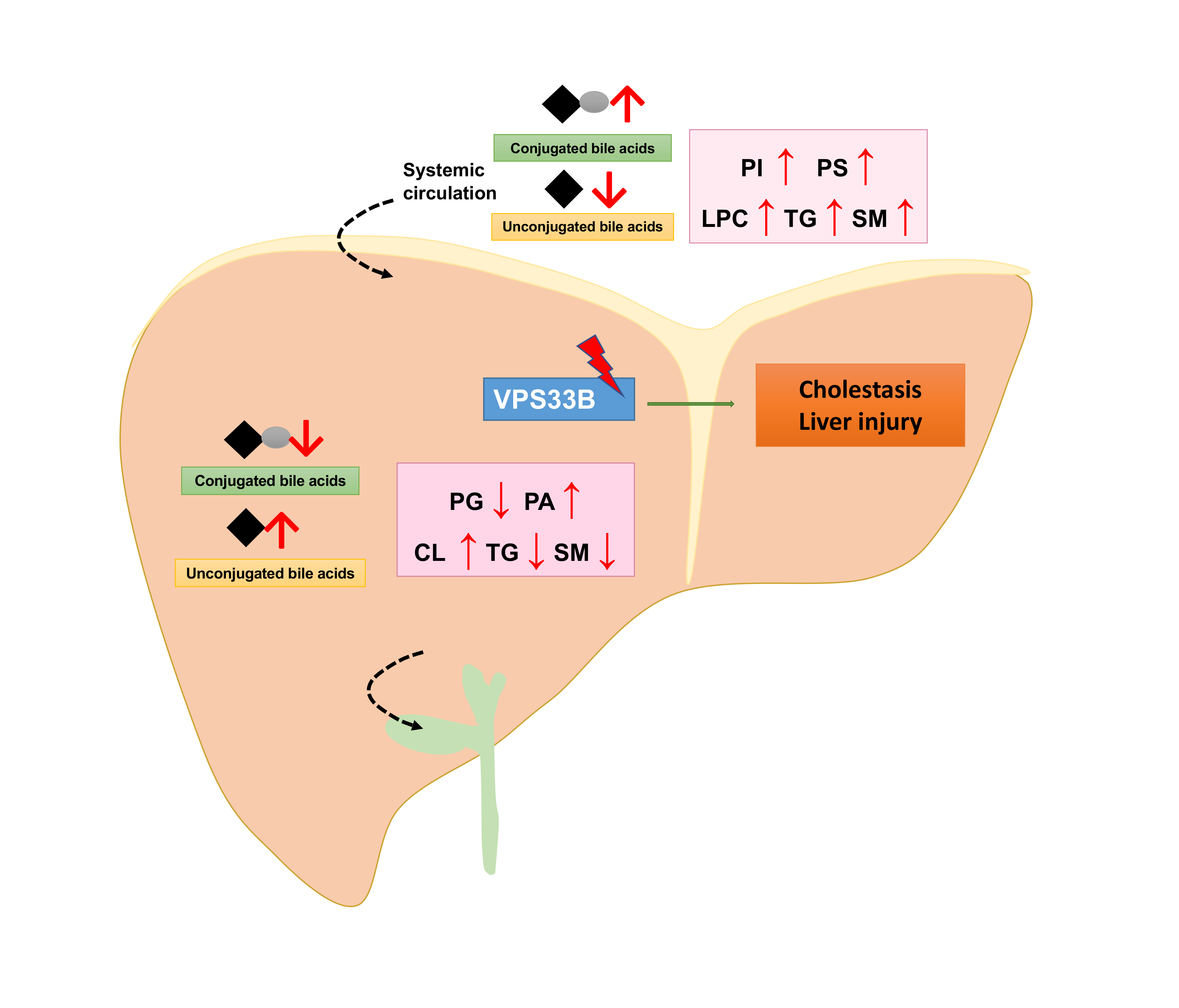

Supplement: Supplementary file 9 [file Image_8.JPEG]
